# Falls and Fractures in the Elderly with Sinus Node Disease: The Impact of Pacemaker Implantation

**DOI:** 10.1155/2012/498102

**Published:** 2012-10-24

**Authors:** Nazmi Krasniqi, Diana Segalada, Thomas F. Lüscher, Kurt Lippuner, Laurent Haegeli, Jan Steffel, Thomas Wolber, Corinna Brunckhorst, Johannes Holzmeister, David Hürlimann, Firat Duru

**Affiliations:** ^1^Clinic for Cardiology, University Hospital Zurich, Rämistrasse 100, B 8091 Zurich, Switzerland; ^2^Center for Integrative Human Physiology, University of Zurich, 8057 Zurich, Switzerland; ^3^Osteoporosis Unit, University Hospital and University of Bern, 3010 Bern, Switzerland

## Abstract

*Background*. Falls and fractures in the elderly are among the leading causes of disability. We investigated whether pacemaker implantation prevents falls in patients with SND in a large cohort of patients. *Methods*. Patient demographics and medical history were collected prospectively. Fall history was retrospectively reconstituted from available medical records. The 10-year probability for major osteoporotic fractures was calculated retrospectively from available medical records using the Swiss fracture risk assessment tool FRAX-Switzerland. *Results*. During a mean observation period of 2.3 years after implantation, the rates of fallers and injured fallers with fracture were reduced to 15% and 6%, respectively. This corresponds to a relative reduction in the number of fallers of 75% (*P* < 0.001) and of injured fallers of 63% (*P* = 0.014) after pacemaker implantation. Similarly, the number of falls was reduced from 60 (48%) before pacemaker implantation to 22 (18%) thereafter (relative reduction 63%, *P* = 0.035) and the number of falls with injury from 22 (18%) to 7 (6%), which corresponds to a relative reduction of 67%, *P* = 0.013. *Conclusion*. In patients with SND, pacemaker implantation significantly reduces the number of patients experiencing falls, the total number of falls, and the risk for osteoporotic fractures.

## 1. Background

Falls and fractures are among the leading causes of disability and loss of independence in the elderly [1].There are a variety of causes for falls, some of which have been related to rhythm disorders such as atrioventricular block [2], carotid sinus hypersensitivity [3, 4], and sinus node disease (SND) [2]. Typical clinical manifestations are syncope, bradycardia, and dizziness. There are data suggesting that pacemaker implantation reduces falls in the elderly suffering from atrioventricular block or carotid sinus hypersensititvity, and hence, it may even decrease healthcare costs [2, 3, 5–7]. On the other hand, such a reduction in falls and healthcare costs has not been documented yet in SND, although pacemaker implantation has been a standard treatment for many years in this patient group [8].

In an attempt to reduce public healthcare costs caused by osteoporotic fractures and the morbidity associated with it, a country-specific fracture risk assessment tool (FRAX available under http://www.shef.ac.uk/FRAX/tool.jsp?country=15) was introduced [9, 10] by analyzing characteristics of patients suffering from osteoporotic fractures. The risk factors that were used to calculate fracture risk in patients were identified to be advanced age, female sex, low weight, previous fracture, among others. By applying this FRAX assessment tool to patients at risk, 10-year probability for both hip fracture and other major osteoporotic fractures can be calculated for each patient individually. Cost-effective intervention thresholds have been established for some countries [11, 12].

FRAX is a fracture risk calculator which was developed by the WHO and calibrated for single countries (more than 50 country versions available to date) based on local fracture epidemiological data. The calculation of the FRAX score requires the availability of a series of risk factors that predict the 10-year probability of experiencing a fracture corrected for remaining life expectancy at a given age. Thus, FRAX is to bone and fracture risk what the PROCAM or the ESC scores are to cardiovascular risk. This score could also be used for highly specific population (such as patients with SND implanted with a pacemaker), because there is no available evidence indicating that their fracture risk should be based on other risk factors than those validated for the general population (age, BMI, sex, fracture history, parental history of hip fracture, current smoking, glucocorticoid intake, rheumatoid arthritis, secondary osteoporosis, excessive alcohol consumption, and bone mineral density measured at the femoral neck). However, FRAX does not assess falls, an important risk factor for fractures. FRAX and falls are complementary dimensions of fracture risk, FRAX reflecting the propensity of bone to fracture and falls representing the inaugural event that may lead to a subsequent fracture, especially in patients with high FRAX scores.

Conceptually, SND patients are at increased risk for fractures and might benefit from therapies aimed at reducing this risk such as pacemaker implantation. Indeed, for cardiovascular disease in general there is evidence for a link to osteoporosis [13, 14]. Whether or not pacemaker implantation prevents falls in patients with SND, however, remains unproven. We therefore aimed to investigate the effects of pacemaker implantation on future fractures in patients with SND using the FRAX tool in a consecutive cohort seen at a single tertiary care center. Specifically, it was the aim of the present analysis to define the characteristics of SND patients, including their fall incidence one year before and after pacemaker implantation. The primary objective was to evaluate and compare the extent of the risk reduction in falls with and without injury. As secondary objectives, we determined the fracture risk profile and the ten-year fracture risk in SND patients based on the validated FRAX algorithm [9, 10].

## 2. Methods

### 2.1. Patients

The electronic patient database of the Clinic for Cardiology of the University Hospital of Zurich, Switzerland, was searched for patients with a diagnosis of SND who were implanted a pacemaker and had follow-ups in the outpatient cardiology setting between January 1, 1996, and December 31, 2009. Patient demographics, general medical history, cardiovascular disease history including surgery and arrhythmia, symptoms, and cardiovascular drug therapy were collected prospectively and analyzed retrospectively. Only patients with a diagnosis of SND who were implanted a pacemaker and had followups in the outpatient cardiology setting between January 1, 1996, and December 31, 2009, were included. Generally the patient's medication was not substantially changed after pacemaker implantation. The diagnosis of SND and the controls were made by Holter ECG and after pacemaker implantation by pacemaker control. The implantation of pacemaker in SND patients and the availability of the data represent all the inclusion criteria. Written consent was given by the patients for their information to be stored in the hospital database and used for research. The cantonal ethics committee of canton Zurich acknoweledged the consent to use the data for the research. 

Table 1 shows the baseline characteristics of the patients included in the study.

### 2.2. Fall History

In our study we used the WHO definition of fall (inadvertently coming to rest on the ground, floor, or other lower level, excluding intentional change in position to rest in furniture, wall, or other objects). The patients were personally interviewed in a standardised manner, using the WHO definiton of fall. Fall history was retrospectively reconstituted from the medical records. The falls after pacemaker implantation were documented by using the same proceeding as before pacemaker implantation (patients personally interviewed using the WHO definition of fall). For fracture falls there was a confirmation by imaging. The 10-year probability for hip and any major osteoporotic fracture was calculated retrospectively from available medical records using the Swiss fracture risk assessment tool FRAX-Switzerland.

### 2.3. Statistical Analysis

Descriptive statistical methods were used to calculate the prevalence of patient characteristics in the SND population (mean, standard deviation). Predictors of falls before pacemaker implantation were identified by an exploratory multiple regression analysis. The incidence and rate of falls with and without injury before and after pacemaker implantation were compared with a paired *t*-test. The level of statistical significance was defined as a two-sided *P* less than 0.05. The following parameters were used in the multiple regression model aimed at identifying predictors of falls during the 12 months before pacemaker implant: age, height, weight, BMI, office-based heart rate, and LVEF; clinical signs and symptoms (syncope, fatigue, dizziness, dyspnea, bradycardia); drug therapy by therapeutic class (anticoagulants, class I and III antiarrhythmics, ACE inhibitors or ARBs, beta-blockers, cardiac glycosides, diuretics, vasodilators); presence of persistent or paroxysmal atrial and/or ventricular arrhythmia; presence of atrioventricular conduction disorders; history of ablation, coronary artery surgery, or valve surgery; history of cardiovascular disease (cardiomyopathy, coronary artery disease, valve dysfunction, stroke, hypertension). All calculations were done with the statistical software StatsDirect version 2.7.8 (StatsDirect Ltd, Altrincham, Cheshire, UK).

## 3. Results

### 3.1. Patient Cohort

A total of 1803 patients underwent pacemaker implantation at our institution. Of these, 164 (9.1%) had SND and were regularly seen for a follow-up as outpatients. Patients implanteding a PM at the University Hospital are usually referred back to their treating cardiologist. However, patients who were not referred to the University hospital by a cardiologist in private practice and those who decided to continue followup directly at the University hospital are those we see on regular basis for followup as outpatients and of whom we have complete records. All these patients had 2 clinical controls combined with pacemaker controls per year. These were included in the analysis. From 164 patients with SND who implanted a PM, only patients aged 40+ were included in the analysis (of which the youngest was 45 years old, 32 patients were >65 years old (23.3%)). Thirteen patients below 40 years of age were excluded. In addition, a 61-year-old “frequent faller” (who presented 80 falls during the year before PM implantation, more than half of all recorded falls) was excluded from the analysis for statistical reasons. Thus 150 patients were included in the baseline characteristics analysis.

**Figure 1:**
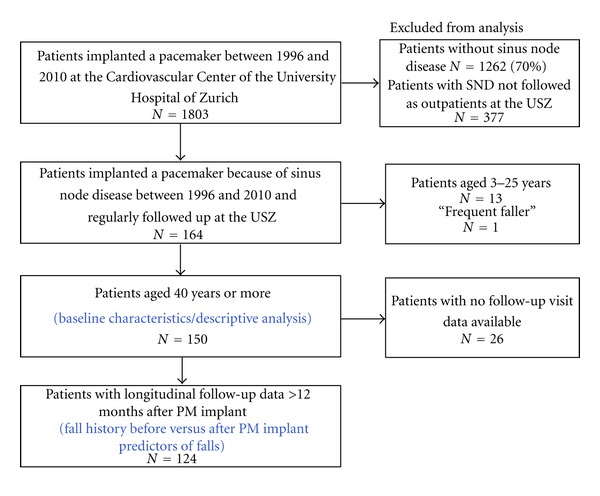
Consort table documenting patient disposition based on inclusion and exclusion criteria.

Additional 26 patients were excluded from the fall analysis, because of missing follow-up data for falls during at least 12 months since pacemaker implantation (Figure 1). Therefore, a total of 150 patients were included in the descriptive analysis for patient characteristics and 124 in the fall history analysis (Table 1).

150 patients (61% male) fulfilling the criteria were included for a descriptive analysis of the baseline characteristics (Figure 1). Mean age at the time of implantation was 71.9 ± 7.9 years. The prevalence of diabetes mellitus was 16.7%. 37.0% of the patients were current or past smokers. All patients had a history of cardiovascular pathology, including coronary artery disease (41.3%), valvular dysfunction (34.7%), and cerebrovascular disease (14.7%). Prevalence of hypertension was high (87.4%) while symptomatic hypertension was rarely observed (3.6%). 55 patients (36.4%) had cardiac interventions, thereof catheter ablation 5.3%, coronary intervention 17.9%, and valve surgery 15.2%.

The patients included were diagnosed to have SND in the presence of the following clinical findings: sinus arrest or sinus pause 44.7%, bradycardia/tachycardia syndrome 54.7%, sinus bradycardia 78.7%, and chronotropic incompetence 10%, which in turn triggered symptoms characteristic for SND: dizziness 39%, syncope 35%, dyspnea 28%, and fatigue 23%.

### 3.2. Medications

All patients were treated with cardiovascular drugs, most frequently anticoagulants including acetylsalicylic acid (78%), followed by angiotensin converting enzyme inhibitors or angiotensin II receptor antagonists (50%), beta-adrenergic receptor blockers (34%), and diuretics (29%). Less commonly used medications were antiarrhythmics including calcium channel blocking agents, vasodilators, and cardiac glycosides.

### 3.3. FRAX Analysis

**Figure 2:**
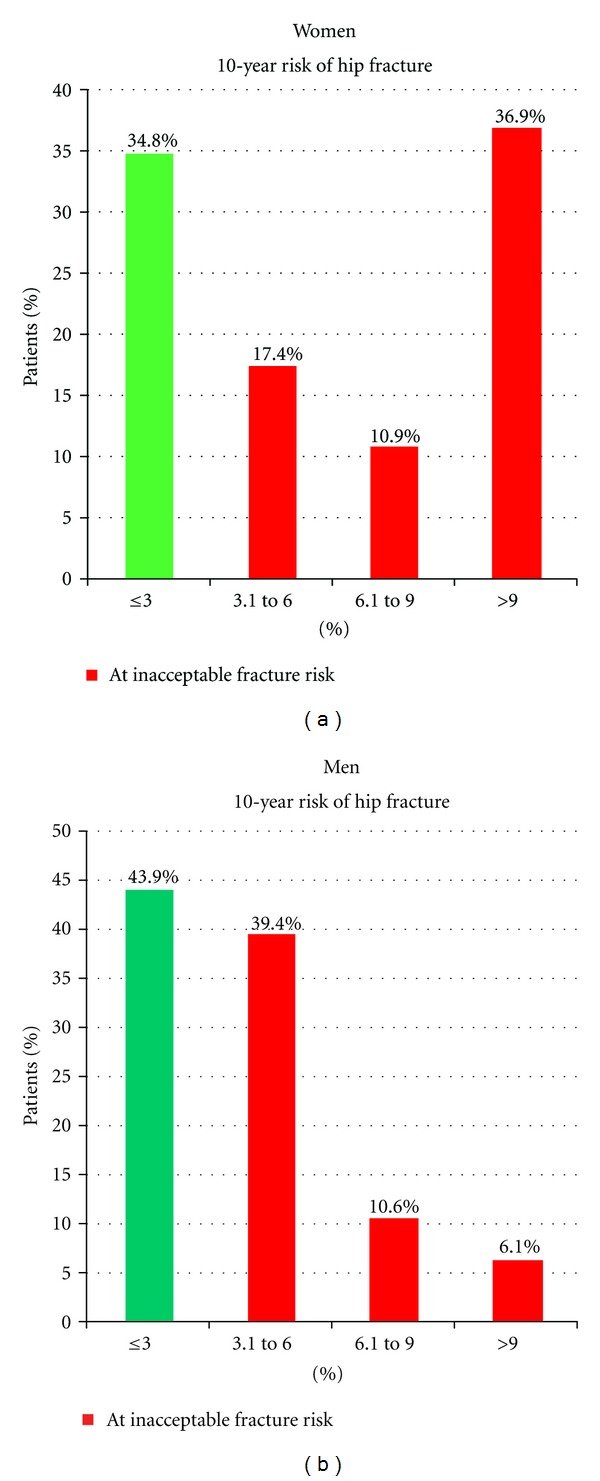
10-year probability for a hip fracture assessed by FRAX in women and men with sinus node disease.

In 134 (89%) patients with SND enough information in patient history to calculate the FRAX score could be gathered, resulting in a mean 10-year risk for hip fracture of 8.7% and 3.7% for women and men, respectively. This corresponds to a twofold increase of hip fracture risk compared to age-, sex-, and BMI-matched individuals without additional risk factors. FRAX analysis also showed that 65% of the female and 56% of the male patients had an individual 10-year probability for hip fracture that was 3.0% or more, therefore exceeding the intervention threshold of 3% defined as cost-effective by the National Osteoporosis Foundation in the USA [11, 12] (Figure 2).

The intervention threshold for the 10-year probability of suffering any osteoporotic fracture in turn was defined as 10% or higher, which was again exceeded by 70% of female and 36% of male patients in our analysis.

### 3.4. Predictors of Falls

Only two statistically significant predictors of falls in SND patients could be identified in the multiple regression analysis: treatment with a diuretic (*r* = 0.26, *P* = 0.005) and history of syncope (*r* = 0.6, *P* < 0.001). The fact that falls are frequent in patients with syncope is not surprising, while with diuretic treatment it can be argued that such patients are more likely to suffer from hypotensive episodes.

### 3.5. Documented Falls

**Figure 3:**
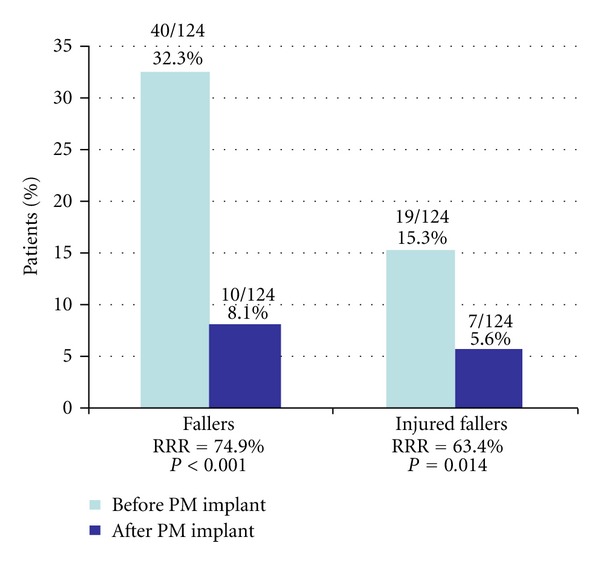
Number of patients experiencing at least one fall before and reduction of falls after pacemaker implantation for sinus node disease. (RRR: relative risk reduction).

Of the 124 patients with available follow-up data, 40 (32%) had experienced at least one fall in the 12 months preceding pacemaker implantation. Of those 19 (47.5% of fallers, 15.3% of total) had acquired an injury while falling. During a mean 2.3 years of followup after pacemaker implantation, the number of falls was reduced to 10 patients (8.1%) falling and 7 (70% of fallers, 5.6% of total) suffering injury while falling. This corresponds to a relative reduction in the number of fallers by 75% (*P* < 0.001) and of injured fallers by 63% (*P* = 0.014; Figure 3).

**Figure 4:**
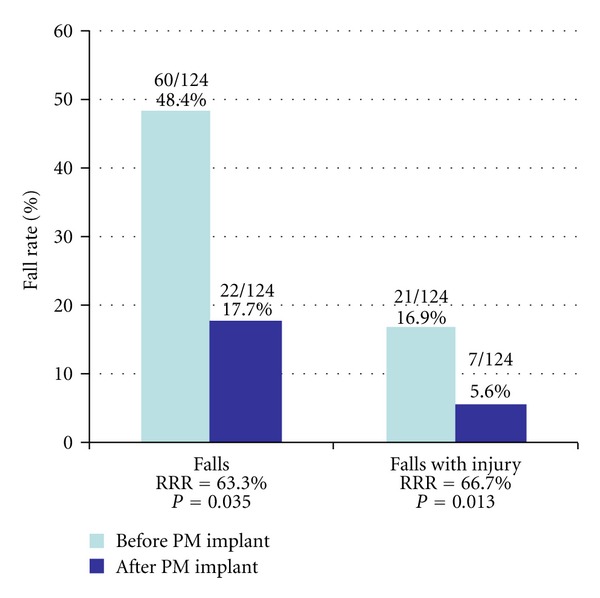
Fall rate before and after pacemaker implantation for sinus node disease. (RRR = relative risk reduction).

Considering the number of falls rather than the number of patients falling, a similar effect could be observed. Indeed, the number of falls was reduced from 60 before pacemaker implantation to 22 thereafter and the number of falls with injury from 22 to 7, which corresponds to a relative risk reduction of 63% (*P* = 0.035) and 67% (*P* = 0.013) respectively (Figure 4).

One patient was excluded from the analysis because he was a “frequent faller.” However, the beneficial effect of pacemaker implantation on the number of falls could also be observed in this particular case, as the patient experienced 80 falls during the 12 months preceding pacemaker implantation, a number which was reduced eightfold to 10 falls during the 12 months following pacemaker implantation.

## 4. Discussion

Our study is the first, to our knowledge, to evaluate the effect of pacemaker implantation on the incidence of falls and fractures which had been diagnosed with SND. Our findings suggest that pacemaker therapy is highly effective in preventing falls and resultant fracture injuries in this patient population who were mostly elderly. Indeed, using a novel statistical analysis we found that during a follow-up period of over 2 years, the implantation of a pacemaker reduced the rates of falls and fracture injuries to 15% and 6%, respectively. This corresponds to a relative risk reduction in the number of fallers of 75% and of injured fallers of 63%. Similarly, the absolute number of falls was reduced from 60 to 22 and the number of falls with injury from 22 to 7. 

Falls are an important problem in elderly patients causing a significant number of unplanned hospitalizations, operations (mainly hip replacement) and invalidity, and eventually nursing-home care [13–15]. Accordingly, the costs of falls are considerable and their causes are numerous [16]. As expected the mean age of the patients included in this study averaged more than 70 years and many of them had considerable comorbidities such as diabetes, valvular heart, coronary artery disease, and/or cerebrovascular disease.

Not all falls are preventable by medical measures, but some are, particularly those related to bradycardias [17]. Pacemakers have been used for this indication ever since their introduction by senning in the late 1950s [18, 19]. The current study, however, documented for the first time the amount of benefit provided by this therapy in the prevention of falls in patients with SND using the Swiss fracture risk assessment tool FRAX-Switzerland. Within the entire registry of 1803 pacemaker implantations at our institution, those for SND amounted to about 10% of all patients.

The World Health Organization (WHO) defined osteoporosis as “a systemic skeletal disease characterized by low bone mass and microarchitectural deterioration of bone tissue, with a consequent increase in bone fragility and susceptibility to fracture” [20]. The most frequent complications of the disease are the typical osteoporotic fractures occurring at the hip, spine, distal forearm, and proximal humerus commonly referred to as major osteoporotic fractures [21]. In 50-year-old Swiss men and women, the remaining lifetime probabilities of presenting a major osteoporotic fracture were 20.2% and 51.3%, respectively [9]. Between years 2000 and 2007, the burden of hospitalized osteoporotic fractures to the Swiss healthcare system has continued to increase in both sexes in Switzerland, driven by an increasing number and incidence of hospitalizations for nonhip fractures, although the incidence of hip fractures has declined [22]. Furthermore, in women, this burden was significantly higher than that of major cardiovascular events (acute myocardial infarction, stroke, and heart failure) and the gap widened over time [23]. Low bone mass, as measured by DXA, is a key predictor of fracture risk [24–27]. However, bone mineral density (BMD) alone does not capture all determinants of fracture probability [28]. Recently, the use of clinical risk factors alone or in combination with BMD has been shown to predict the probability of hip and osteoporotic fractures in men and women [29]. In order to identify the major clinical risk factors for osteoporotic fracture, the data from nine prospective primary cohorts and 11 prospective validation cohorts, including more than 275,000 persons corresponding to 1.4 million person-years with more than 22,711 reported fractures, were analysed [29]. The validation analysis included the results from the Swiss SEMOF cohort [30]. In addition to any prior fragility fracture that occurred after age 50, age, sex, body mass index, and additional risk factors were considered. These included prior use of glucocorticoids, secondary osteoporosis, rheumatoid arthritis, a parental history of hip fracture, current cigarette smoking, and alcohol intake of three or more units/day. These factors were identified as clinical predictors of osteoporotic fracture probability, independently of BMD [29]. Taking into account local epidemiological data, the impact of these risk factors on the 10-year absolute probability of having a fracture can allow for country-specific prediction of individual fracture probability, based on the individual risk factor profile. This case-finding algorithm, known as FRAX (http://www.shef.ac.uk/FRAX/), has been developed in collaboration with the WHO and has been customized to the epidemiology of several countries including Switzerland [10].

In the present analysis, most of the patients who underwent pacemaker implantation for SND were at increased risk for major fractures, as assessed by their FRAX score. Pacemaker implantation significantly decreased the fall rate and thereby the number of initial events generally required for a subsequent fracture.

## Figures and Tables

**Table 1:** Baseline characteristics of included patients.

	Mean	SD	Range (min–max)
Age at PM implantation (years)	71.9	9.7	45–94
LVEF (%)	55.9	13.3	20–77
Height (cm)	168.8	10.5	142–195
Weight (kg)	73.5	13.9	42.5–111
BMI	25.7	3.7	17.1–38.7
Heart rate at rest (bpm)	66.8	25.3	28–180
FRAX available (*N* = 134)			
10-year risk of hip fracture	**5.5**	**7.0**	**0.1–58**
10-year risk of major osteoporotic fractures	**14.5**	**10.6**	**2.9–64**

	*N *		%

Total 40+	**150**		100.0
Male sex	91		60.7
Diabetes mellitus	25		16.7
Ever smoked	56		37.3
NYHA class			
No heart failure	46		30.7%
NYHA I	39		26.0%
NYHA II	37		24.7%
NYHA III	22		14.7%
NYHA IV	6		4.0%
