# Corrigendum to “Falls and Fractures in the Elderly with Sinus Node Disease: The Impact of Pacemaker Implantation”

**DOI:** 10.1155/2017/3297515

**Published:** 2017-10-19

**Authors:** Nazmi Krasniqi, Diana Segalada, Thomas F. Lüscher, Kurt Lippuner, Laurent Haegeli, Jan Steffel, Thomas Wolber, Corinna Brunckhorst, Johannes Holzmeister, David Hürlimann, Firat Duru

**Affiliations:** ^1^Clinic for Cardiology, University Hospital Zurich, Rämistrasse 100, B 8091 Zurich, Switzerland; ^2^Center for Integrative Human Physiology, University of Zurich, 8057 Zurich, Switzerland; ^3^Osteoporosis Unit, University Hospital and University of Bern, 3010 Bern, Switzerland

In the article titled “Falls and Fractures in the Elderly with Sinus Node Disease: The Impact of Pacemaker Implantation” [1], there was an error regarding the FRAX® tool, which should be clarified as follows:

The article notes: “FRAX is a fracture risk calculator which was developed by the WHO and calibrated for single countries (more than 50 country versions available to date) based on local fracture epidemiological data.” However, the World Health Organization (WHO) did not develop, test, or endorse the FRAX tool or its recommendations [2]. The metabolic bone disease unit at the University of Sheffield that developed FRAX was a WHO Collaborating Centre from 1991 to 2010, but treatment guidelines must undergo a formal process before they can be endorsed by the WHO.

## References

[1] Krasniqi N., Segalada D., Lüscher T. F. (2012). Falls and fractures in the elderly with sinus node disease: the impact of pacemaker implantation. *Cardiology Research and Practice*.

[2] Ford N., Norrisb S. L., Hill S. R. (2016). Clarifying WHO’s position on the FRAX® tool for fracture prediction. *Bulletin of the World Health Organization*.

